# Prediction and visualization data for the interpretation of sarcomeric and non-sarcomeric DNA variants found in patients with hypertrophic cardiomyopathy

**DOI:** 10.1016/j.dib.2016.03.004

**Published:** 2016-03-10

**Authors:** Irene Bottillo, Daniela D’Angelantonio, Viviana Caputo, Alessandro Paiardini, Martina Lipari, Carmelilia De Bernardo, Silvia Majore, Marco Castori, Elisabetta Zachara, Federica Re, Paola Grammatico

**Affiliations:** a Medical Genetics, Department of Molecular Medicine, Sapienza University, San Camillo-Forlanini Hospital, Rome, Italy; b Department of Experimental Medicine, Sapienza University of Rome, Rome, Italy; c Department of Biochemical Sciences, Sapienza University of Rome, Rome, Italy; d Cardiomyopathies Unit, Division of Cardiology and Cardiac Arrhythmias, San Camillo-Forlanini Hospital, Rome, Italy

## Abstract

Genomic technologies are redefining the understanding of genotype–phenotype relationships and over the past decade, many bioinformatics algorithms have been developed to predict functional consequences of single nucleotide variants. This article presents the data from a comprehensive computational workflow adopted to assess the biomedical impact of the DNA variants resulting from the experimental study “Molecular analysis of sarcomeric and non-sarcomeric genes in patients with hypertrophic cardiomyopathy” (Bottillo et al., 2016) [1]. Several different independently methods were employed to predict the functional consequences of alleles that result in amino acid substitutions, to study the effect of some DNA variants over the splicing process and to investigate the impact of a sequence variant with respect to the evolutionary conservation.

**Specifications Table**

**Table:** 

Subject area	Biology
More specific subject area	In silico predictions of DNA variants
Type of data	Tables, figures
How data was acquired	Prediction tools: SIFT, Polyphen HDIV, Polyphen HVAR, Provean, LRT, Mutation Taster, Mutation Assessor, FATHMM, RadialSVM, LR, CADD, HSF, GERP++, PhyloP placental, PhyloP veterbrate, SiPhyMolecular Modeling
Data format	Processed, filtered and analyzed
Experimental factors	Genomic DNA from peripheral blood was tested by next generation sequencing on Ion Torrent PGM (ThermoFisher, Carlsbad, CA, USA) with a custom cardiomyopathy panel
Experimental features	The identified rare (Minor Allele Frequency ≤0,01) non-synonymous DNA changes were subjected to different *in silico* predictions
Data source location	Rome, Italy
Data accessibility	These data are with this article

**Value of the data**

• These data delineate a prompt informatic pipeline for the prioritization of the most likely pathogenetic DNA variants in a clinical context.
• These data are supportive for the researchers to evaluate the prevalence of sarcomeric and non-sarcomeric gene variants in hypertrophic cardiomyopathy.
• The described computational strategy is helpful to researchers for the rapid interpretation of Variants of Unknown Significance (VUS) implicated in rare, common and complex diseases.

**1. Data**

Here we report the in silico predictions data of the non-synonymous changes found in 41 HCM patients and in 3 HCM-related cases [1] (Table 1).

## 2 Experimental design, materials and methods

### 2.1 Analysis of the nucleotides׳ evolutionary conservation

Nucleotide-specific estimates of evolutionary constraint were explored by (i) GERP++ (Genomic Evolutionary Rate Profiling); (ii) PhyloP placental; (iii) PhyloP veterbrate and (iv) SiPhy.

### 2..2 Analysis of the splicing variants

The analysis of intronic variants leading to splicing defects was tested by Human Splicing Finder (HSF) 3.0.

### 2.3 Analysis of the missense variants

The effect of missense changes on the structure and function of a human protein was predicted by: (i) SIFT (Sorting Intolerant From Tolerant), (ii) PolyPhen-2 (Polymorphism Phenotyping v2) HDIV, that identifies human damaging mutations by assuming differences between human proteins and their closely related mammalian homologs as non-damaging; (iii) PolyPhen-2 HVAR, that identifies human disease-causing mutations by assuming common human nsSNPs as non-damaging; (iv) Provean (Protein Variation Effect Analyzer); (v) LRT (Likelihood Ratio Test) that identifies conserved amino acid positions and deleterious mutations using a comparative genomics data set of multiple vertebrate species; (vi) Mutation Taster; (vii) Mutation Assessor; (viii) FATHMM (Functional Analysis through Hidden Markov Models); (ix) RadialSVM (Radial Support Vector Machine); (x) LRT (Logistic Regression Test); (xi) CADD v1.3 (Combined Annotation–Dependent Depletion), a method for objectively integrating many diverse annotations into a single measure (C score) for each variant; and (xii) molecular modeling.

**Fig. 1:**
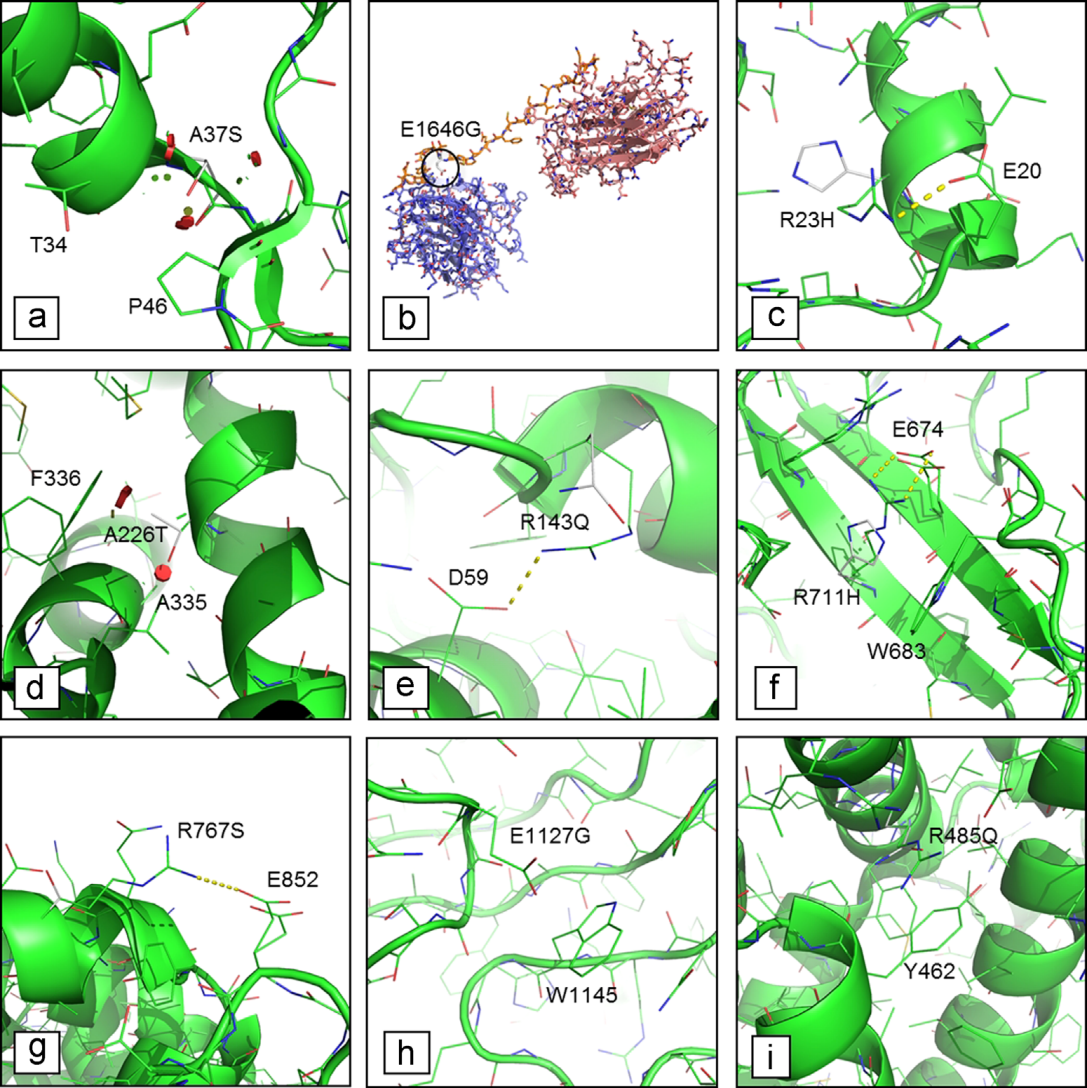
Structural comparison of wild-type and mutant forms for (a) FLH2 A37S; (b) LAMA4 E1646G; (c) MYH6 R23H; (d) MYH7 A226T; (e) MYH7 R143Q; (f) MYOM1 R711H; (g) PKP2 R767S; (h) RYR2 E1127G; (i) RYR2 R485Q. The mutation is indicated in white. The predicted structural effects of mutations are: (a, d) steric hindrance (red circles); (b) local misfolding of linker domain (orange); (c, e, f, g) loss of important inter-residues contacts; (h) loss of a π-anion interaction; (i) loss of a π-cation interaction.

**Fig. 2:**
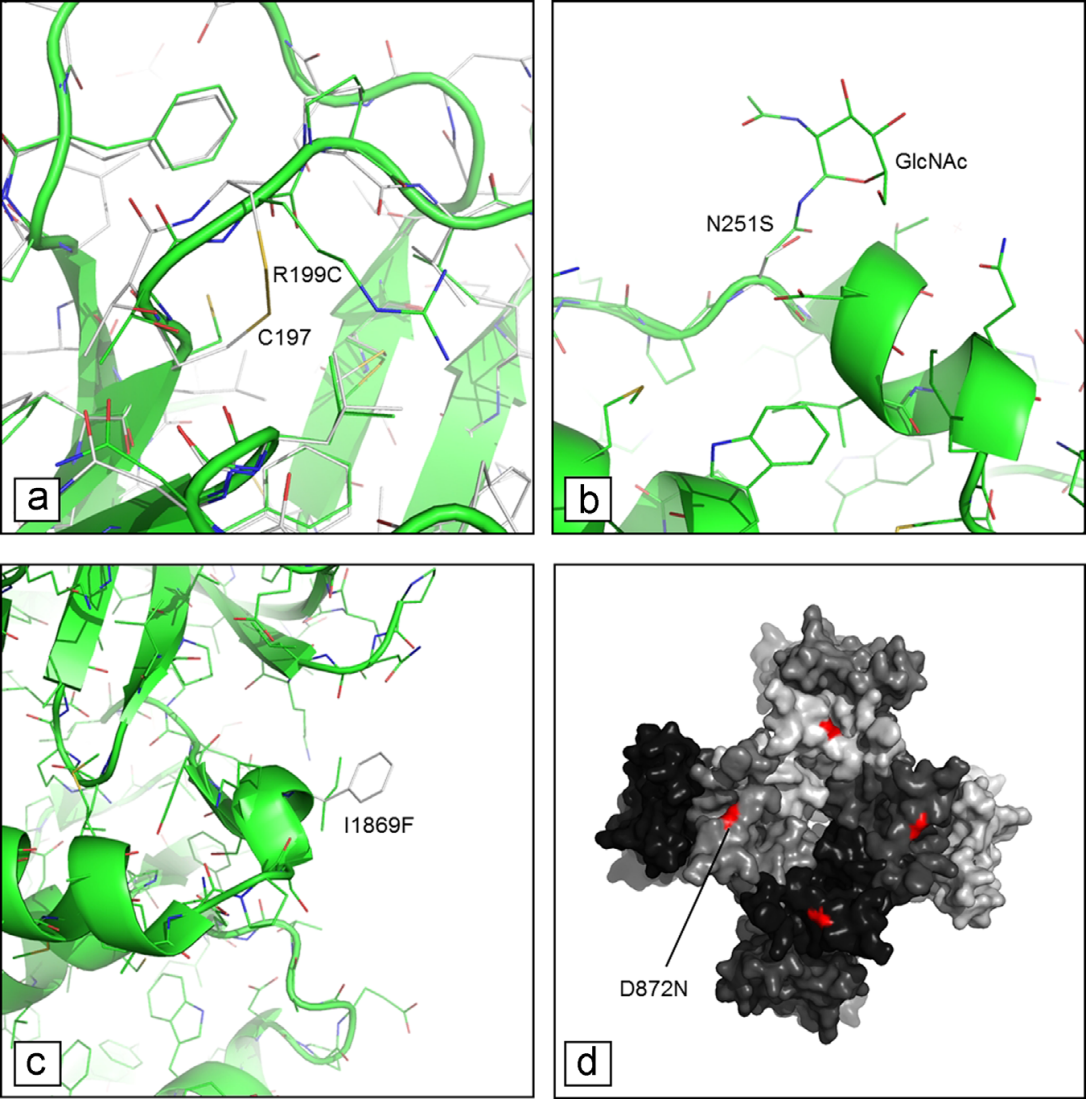
Effects of nsSNVs for: (a) the cadherin domain of DSC2. The mutant R199C in the cadherin domain of DSC2 is predicted to introduce a disulfide bond with the near Cys197 residue (Cα-Cα distance ~6 Å), and possibly to result in local misfolding of the cadherin domain; (b) the melibiase domain of GLA. Mutant N215S of the melibiase domain of GLA results in the loss of a glycosylated site probably affecting the protein structure and/or function; (c) the FGF13 interaction domain of SCN5. Mutation I869F localizes on a solvent-exposed hydrophobic path of the domain of interaction with fibroblast growth factor 13 (FGF13). The I869F mutation could affect the recognition of the FGF13 protein; (d) the Na-Channel of SCN5. The mutant D872N results in the loss of a negative charge that is approximately located at the Na-channel domain of SCN5, probably affecting cations conductance of the channel. The approximate position of the negatively charged Asp872 residue is shown in red, in each of the four protein subunits forming the channel.

Regarding the molecular modeling, protein structure were experimentally determined by X-ray crystallography, or were inferred by homology modeling means (i.e., availability of a structural template with percentage of identity > 20%). Protein models were built using the homology modeling approach implemented in modeler-9 package [2]. PSI-BLAST was used to find suitable structural templates for each sequence to model [3]. The sequences of each protein target to model and its structural template were then aligned by using the program CLUSTALW [4] and manually manipulated to optimize the matching of several characteristics, including the observed and predicted secondary structural elements, the hydrophobic regions in the three-dimensional structures, the structurally and functionally conserved residues, and *indel* regions in the structures. Then, ten different models were built for each target protein and evaluated using several criteria. The model displaying the lowest objective function [5], which measures the extent of violation of constraints from the structural templates, was taken as the representative model. Superimposition and root-mean-square deviation (RMSD) calculation of Cα traces of the 10 models were performed to detect the most variable and therefore less reliable modeled regions. These invariably corresponded to loop elements. Procheck [6] was used to monitor the stereochemical quality of the representative models, whereas ProsaII [7] was used to measure the overall protein quality in packing and solvent exposure. Mutations on protein structures was carried out using the “Mutate model” script implemented in modeler-9 package [2]. The script takes as input a given three-dimensional structure of a protein (experimentally determined or predicted), and mutates a single residue. The residue sidechain׳s position is then optimized by energy minimization and refined by molecular dynamics simulations. Prediction of protein stability upon mutation was carried out using the DUET server [8]. Sequence identity between the modeled domain and its closest template ranged from 23% (Laminin G-like domain of LAMA4), to nearly 95% (N-terminal globular head domain of VCL). However, in spite of the low value of sequence identity measured in some cases, all of the models resulted in a good overall quality (Prosa Z-score <−2.00), except for CALR3 and SCN5. Given the short length of the predicted PB035848 domain of CALR3 (residues 294-347) and its sequence identity with its template (61%), the measured Prosa Z-score (−1.93) nonetheless indicated a model of quality comparable to a Nuclear Magnetic Resonance (NMR) structure [7] ( Fig. 1, Fig. 2.

## Figures and Tables

**Table 1:** Results of the in silico predictions of the non-synonymous changes found in 41 HCM patients and in 3 HCM-related cases. Deleterious predictions are in bold.




## References

[1] Bottillo I., D׳Angelantonio D., Caputo V., Paiardini A., Lipari M., De Bernardo C., Giannarelli D., Pizzuti A., Majore S., Castori M., Zachara E., Re F., Grammatico P. (2016). Molecular analysis of sarcomeric and non-sarcomeric genes in patients with hypertrophic cardiomyopathy. Gene.

[2] Eswar N., Webb B., Marti-Renom M.A., Madhusudhan M.S., Eramian D., Shen M.Y., Pieper U., Sali A., Baxevanis Andreas D. (2006). Comparative protein structure modeling using modeller. Current Protocols in Bioinformatics.

[3] Friedberg I., Kaplan T., Margalit H. (2000). Evaluation of PSI-BLAST alignment accuracy in comparison to structural alignments, protein science: a publication of the protein. Society.

[4] Thompson J.D., Higgins D.G., Gibson T.J. (1994). CLUSTAL W: improving the sensitivity of progressive multiple sequence alignment through sequence weighting, position-specific gap penalties and weight matrix choice. Nucleic acids Res..

[5] Burke D.F., Deane C.M., Nagarajaram H.A., Campillo N., Martin-Martinez M., Mendes J., Molina F., Perry J., Reddy B.V., Soares C.M., Steward R.E., Williams M., Carrondo M.A., Blundell T.L., Mizuguchi K. (1999). An iterative structure-assisted approach to sequence alignment and comparative modeling. Proteins.

[6] Laskowski R.A., Rullmannn J.A., MacArthur M.W., Kaptein R., Thornton J.M. (1996). AQUA and PROCHECK-NMR: programs for checking the quality of protein structures solved by NMR. J. Biomol. NMR.

[7] Sippl M.J. (1993). Recognition of errors in three-dimensional structures of proteins. Proteins.

[8] Pires D.E., Ascher D.B., Blundell T.L. (2014). DUET: a server for predicting effects of mutations on protein stability using an integrated computational approach. Nucleic Acids Res..

