# Supplementary material for: Prediction and visualization data for the interpretation of sarcomeric and non-sarcomeric DNA variants found in patients with hypertrophic cardiomyopathy
Source: Data Brief. 2016 Mar 10;7:607–13. doi: 10.1016/j.dib.2016.03.004 (PMC4802523; doi:10.1016/j.dib.2016.03.004)
Supplement: Supplementary file 1 — Supplementary material [file mmc1.doc]

*Data article*

**Title:**

**Prediction and visualization data for the interpretation of sarcomeric and non-sarcomeric DNA variants found in patients with hypertrophic cardiomyopathy**

**Authors:**

Bottillo Irene^a^, BSc, PhD*, D’Angelantonio Daniela^a^, BSc, Caputo Viviana^b^ BSc, PhD, Paiardini Alessandro^c^ BSc, PhD, Lipari Martina^a^, BSc, De Bernardo Carmelilia^a^, BSc, Majore Silvia^a^, MD, PhD, Castori Marco^a^, MD, PhD, Zachara Elisabetta^e^, MD, Re Federica^e^, MD, Grammatico Paola^a^, BSC, PhD

**Affiliations:**

*^a^Medical Genetics, Department of Molecular Medicine, Sapienza University, San Camillo-Forlanini Hospital, Rome, Italy; ^b^Department of Experimental Medicine, Sapienza University of Rome, Rome, Italy; ^c^Department of Biochemical Sciences, Sapienza University of Rome, Rome, Italy; ^d^Cardiomyopathies Unit, Division of Cardiology and Cardiac Arrhythmias, San Camillo-Forlanini Hospital, Rome, Italy*

**Contact email:**

Irene Bottillo, PhD

Medical Genetics, Department of Molecular Medicine, Sapienza University, San Camillo-Forlanini Hospital

Circonvallazione Gianicolense, 87 - 00152 Rome, Italy

Phone: +39-06-58704622

Fax: +39-06-5870-4657

email: [i.bottillo@gmail.com](mailto:i.bottillo@gmail.com)

**CONFLICT OF INTEREST:** none
